# Stable osteosynthesis of cage in cage technique for surgical treatment of proximal humeral fractures

**DOI:** 10.1186/s12893-021-01235-x

**Published:** 2021-05-04

**Authors:** Jiantao Li, Yuan Gao, Caixia Yin, Hao Zhang, Shaobo Nie, Hui Guo, Chenliang Quan, Hua Chen, Wei Zhang

**Affiliations:** 1 Department of Orthopaedics, Chinese PLA General Hospital, No. 28 Fuxing Road, Beijing, 100853 China; 2 National Clinical Research Center for Orthopedics, Sports Medicine and Rehabilitation, Beijing, China; 3 Department of Nursing, The First Medical Center of Chinese PLA General, Beijing, China; 4 Anesthesia and Operation Center, The First Medical Center of Chinese PLA General, Beijing, China

**Keywords:** Locking compression plate, Peek cage, Proximal humeral fractures, Medial support

## Abstract

**Background:**

The treatment of a displaced proximal humeral fracture is still a matter of controversy. The purpose of this study was to report outcomes at a long-term follow-up after fixation augmentation using peek (polyether-ether-ketone) cage and locking compression plate (LCP).

**Methods:**

A total of 27 patients (average age 53.8 years, range 19–86 years) were treated with peek cage and LCP. All of them had a minimum radiographic and clinical follow-up of 1 years. Outcomes were assessed using the Constant-Murley score (CMS), disability of the arm, shoulder and hand (DASH) score. Complications were also recorded during follow-up.

**Results:**

The average follow-up was 28 months (range 12–48 months). The mean functional outcomes were as follows: CMS, 73.3 (range 61–86); DASH, 45.9 (range 27–68). A total of 4 patients had complications: osteonecrosis developed in one patient, loss of reduction was observed in 1 patient and stiffness was occurred in two patients.

**Conclusion:**

The use of peek cage and LCP has been a valuable option in the treatment of proximal humeral fractures. The complication rate was acceptable. Suitable void filler in the proximal humerus for reconstructing the medial column integrity attains mechanical stability in reducing the incidence of the complications.

**Supplementary Information:**

The online version contains supplementary material available at 10.1186/s12893-021-01235-x.

## Background

Proximal humeral fractures account for 5–6% of all adult fractures [1], and the majority occur in elderly individuals [2]. Displaced proximal humeral fractures require surgical treatment in order to achieve fracture stability and allow for early motion. But surgical management still remains difficult because intraoperative reduction and fixation stability sometimes are unpredictable.

Although biomechanical data and clinical outcomes of LCP have shown promise in treating displaced fractures in general, complication rates of 49% have been reported, and varus malunion and screw perforation are the most two common complications [3, 4]. Hardeman et al. [5] noted worst results in the significantly displaced varus articular fracture in the older patient. Recent studies have demonstrated the importance of reduction and mechanical support of medial column in fracture fixation. Gardner et al. [6] has shown that fractures without medial buttress obtained, even though with anatomic reduction and with screws inferomedial-humeral-head fixation, suffered 29% rate of reduction loss and screw penetration. Accordingly, an intramedullary fibular allograft has been used as an adjunct to the locking plate to provide supplementary medial support in proximal humeral fractures with medial cortex comminution [7, 8]. However, complications of humeral head varus occurred in some related reports [9].

**Fig. 1:**
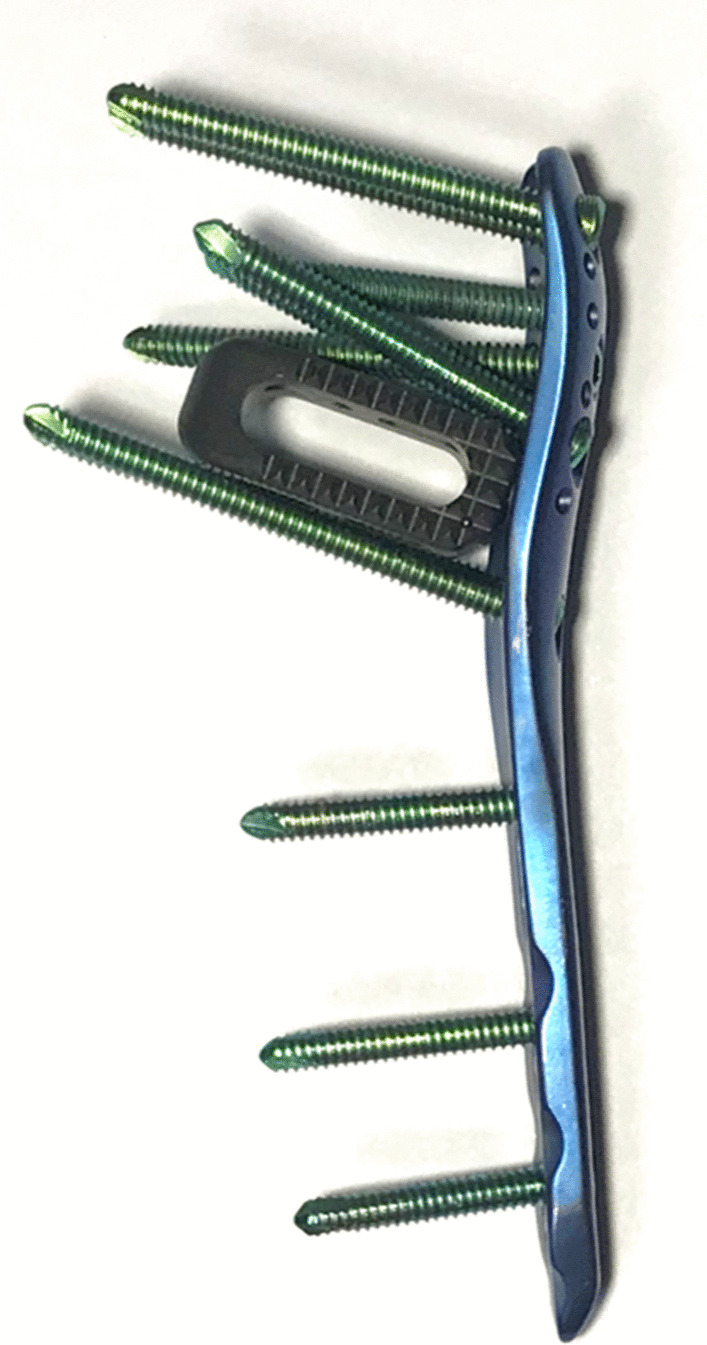
Peek cage locates in a cage space formed by locking screws of LCP

In this study, we present a novel technique and clinical experience using a peek cage to fill the cavity under the humeral head and to provide a medial structural buttress in the treatment of displaced proximal humeral fractures. Peek cage locates in a “cage space” formed by locking screws of LCP (Fig. 1). The purpose of this study is to report outcomes during a period between 1 and 4 years follow-up after surgical treatment with emphasis on the complication rate and function results.

## Methods

This study has been reported in line with the PROCESS criteria [10].

During the period of June 2016 and June 2019, patients with displaced proximal humeral fractures were treated by LCP with peek cage at our hospital. In all of the cases, standard anteroposterior radiograph and computed tomography (CT) scan were performed before surgery. The fractures were classified using the Neer classification.

The proposed operation was offered to medically fit patients with 2–4-part displaced proximal humeral fracture with or without fracture-dislocation. Medial cortex comminution, disengagement of the head from the shaft, or severe angular deformity < 90° or > 160° of the humeral head with respect to the shaft were used as indications for surgery.

We excluded patients with an isolated tuberosity fracture, a pathological fracture, bilateral fracture, previous shoulder injury, multiple injuries, or serious nervous or vascular injury, as well as those presenting beyond 4 weeks after injury.

This research was approved by the Institutional Ethical Committees of the hospital. Each patient signed the informed consent to publish the information/image(s) in an online open-access publication.

### Operative protocol

**Fig. 2:**
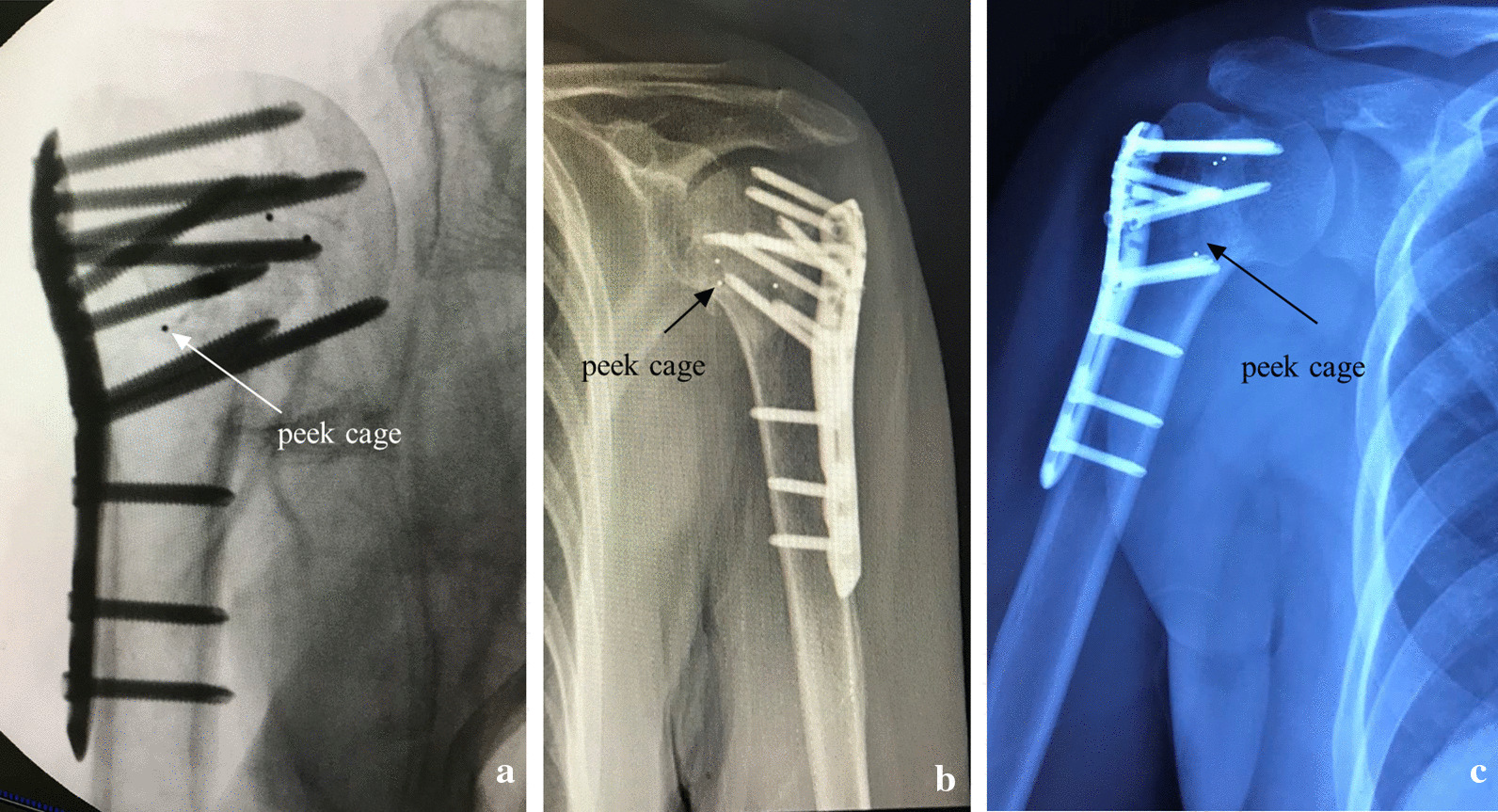
Examples of different peek cage locations to complete the medial reduction and providing medial buttress. **a** Implantation of the cage with a tilt angle to the humeral head. **b** Peek cage with a more horizontal and lower position. **c** Peek cage with vertical position to the proximal fragment

All patients are placed in a beach chair position on a radiolucent operating table. A standard deltopectoral approach is performed. With the axillary nerve identified and protected, the lateral fracture lines are exposed. Nonabsorbable sutures are placed in the supraspinatus, infraspinatus, and subscapularis tendons to allow for traction and reduction of the tuberosity and humeral head fragments. Then, a laminar spreader is put into the intramedullary canal through a lateral cortical window of tuberosity fracture to help keep alignment of the humeral head and shaft. The main goal of the initial operation is to focus on the reduction of the medial cortical column of the proximal humerus. K-wires could be used to provisionally stabilize the reduction. An approximately 2–4 cm length of the peek cage (Concorde, Synthes, Switzerland) is inserted through the lateral fracture window into the medial site of the humeral head fragment as both an indirect reduction tool and mechanical support for the prevention of varus displacement and deformity of the humeral head. Fractures with high-energy mechanisms and medial comminution, or those with osteoporotic bone and persistent medial malalignment following reduction maneuvers, may both be indications for cage augmentation. The cage is inserted to a depth such that the approximate midpoint of the cage is to support effect for humeral head, greater tuberosity, and medial cortical bone. In order to foster osteointegration, bovine cancellous xenogenous bone granules are packed into the cavity and around the peek cage. After that, the rotator cuff sutures are passed through the holes in the head of LCP (Synthes, Switzerland). The LCP is placed between 5 and 10 mm lateral to the bicipital groove and 15–20 mm inferior to the vertex of the humerus head. At this point, anteroposterior (AP) and lateral fluoroscopic views are obtained to ensure that the cage and plate are in the proper position. Head-locking screws are placed in the subcortical bone and distal screws are placed in the shaft. Proximal screws also work to hinge the cage of the humeral surgical neck, maintain the cage into the inferior humeral head, potentially completing the medial reduction and providing medial buttress (Fig. 2). The rotator cuff sutures that were previously placed through the suture holes in the plate are tied to complete the fixation. After careful irrigation, a negative suction drain is placed in the wound followed by layer closure.

In 3-part fractures, we use the same technique with the sutures through the tendons, aiding manipulation, reduction, and temporary fixation fractures. The space under the head is filled with peek cage and bovine cancellous xenogenous bone granules allocated with an inferior vertex in the shaft and the larger base along the internal surface of the calcar. Subsequently, sutures placed through the insertions of each rotator cuff tendon increase stability, and should be used as well as the plate and screws.

In 4-part fractures, we insert sutures into the subscapularis tendon, the supraspinatus tendon, and infraspinatus tendon to provide anchors for reduction, and temporary fixation of the greater and lesser tuberosities. A laminar spreader is put into the intramedullary canal through a lateral cortical window of tuberosity fracture to help keep alignment of the humeral head and shaft. Once the proper correspondence between the head and diaphyseal fracture lines was identified, the peek cage and bovine cancellous xenogenous bone granules are inserted through the lateral fracture window into the medial site of the humeral head fragment. Then attach and fix the plate to the proximal humerus. Sufficient calcar support (screws) is necessary to resist recurrent varus deformity.

### Postoperative care

Postoperatively, patients were immobilized in a sling, and immediate passive mobilization and pendulum exercises were encouraged 1 time per day. In addition, supervised physiotherapy was carried out and gradually ceased around 3 weeks, including a standard protocol of non-weightbearing exercise, active-assisted range of motion, and gentle passive range of motion. The forward elevation and abduction were limited to 100°, and external rotation was limited to 30°.

### Outcome assessment

Patients were clinically followed up which included examination of routine radiographs (AP view and “Y” view) to evaluate the quality of reconstruction, healing, and possible necrosis.

The “humeral head height” between the superior edge of the humeral head and the top edge of the proximal plate was measured on AP radiographs of the shoulder, postoperatively, and at last follow-up. A decrease of the height > 5 mm was interpreted as a loss of reduction. The humeral neck-shaft angle was measured as Agudelo’s description [11].

Patients were assessed using the Constant-Murley score (CMS), disability of the arm, shoulder and hand (DASH) score. Complications, such as loss of reduction, varus malunion, screw perforation, infection, and humeral head necrosis, were recorded during the follow-up.

### Statistical analysis

A descriptive analysis of the data was performed by SPSS 22.0 software, with average values and the associated 95% confidence intervals (CIs) and ranges reported. Complication and functional data were stratified by age groups: young adult (18–44 years old); adult (45–59 years old); elderly (≥ 60 years old). We used the Chi-square and Fisher’s exact tests to analyze the categorical variables between age groups in terms of complication data. The Kruskal–Wallis test was used to compare the groups of functional data. P < 0.05 was considered statistically significant.

## Results

**Fig. 3:**
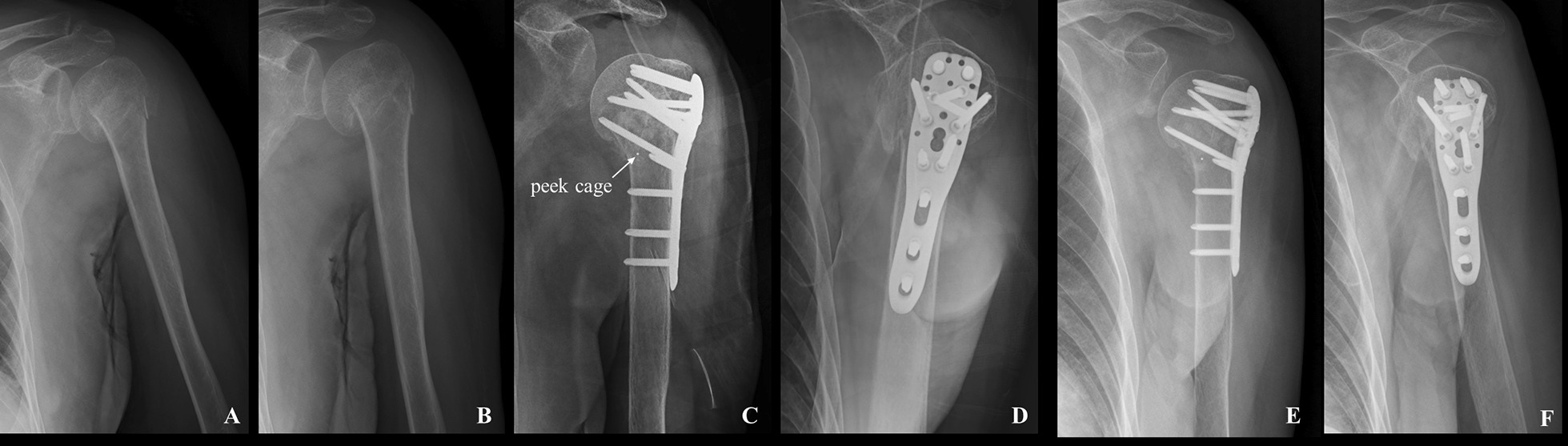
Radiograph of a 47-year-old woman with 3-part fractures. **a**, **b** Preoperative X-ray film; **b**, **c** Peek cage was used as volumetric filling in the medial site to prevent the humeral head collapse, facilitating reduction, and to provide mechanical support; **e**, **f** X-ray film 3 months after the operation

**Fig. 4:**
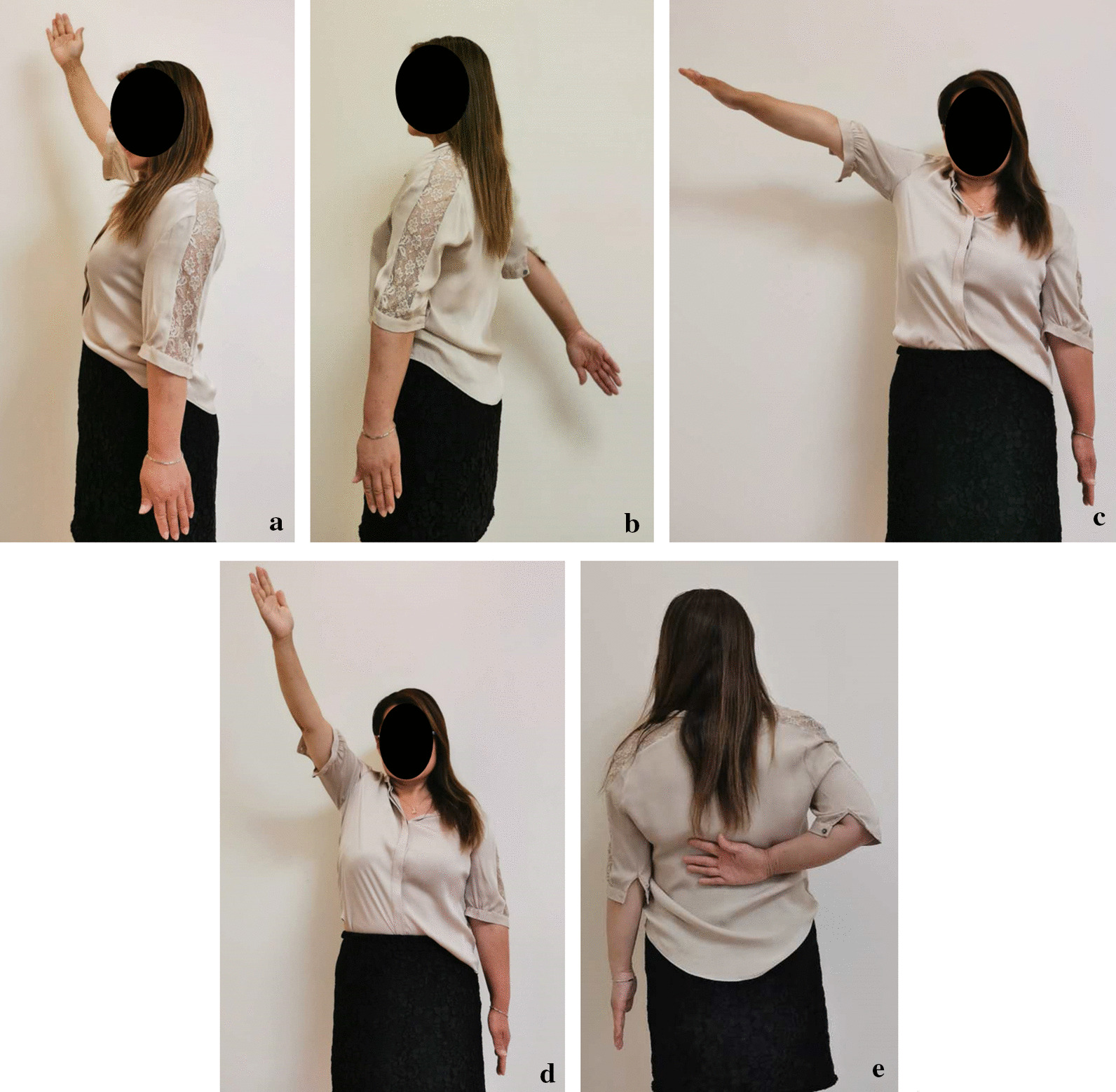
Function recovery of the patient in Fig. 3 at follow-up period

Twenty seven patients were included, in which all patients had an early follow-up of 6 weeks and a minimum follow-up of 12 months [average follow-up, 28 months (95% CI 23–33 months); range 12–48 months]. There were 14 males and 13 females. 21 (77.8%) cases were with the dominant arm. 18 patients suffered from medial comminution. The mean angle of the postoperative humeral neck-shaft was 128.71 degrees. According to the postoperative humeral neck-shaft angle, there were 23 patients with anatomical reduction, 3 patients with acceptable reduction and 1 patient with malreduciton. More details were listed in Table 1 and shown in Figs. 3, 4.

**Table 1:** Patient Demographic Data

Variables	Total Group (no. [%])	Mean Age (Range)(yr)
All patients	27	53.8 (19–86)
Male	14 (51.9)	55.6 (19–83)
Female	13 (48.1)	51.9 (21–86)
Injury mechanism		
Fall	18 (66.7)	51.8 (19–86)
Vehicle accident	9 (33.3)	57.9 (25–83)
Occupation		
Sedentary work	6 (22.2)	38.2 (24–68)
Manual work	5 (18.5)	42.2 (34–65)
Not working/retired	16 (59.3)	63.3 (19–86)
Neer fracture classification		
2-part	11 (40.7)	56.0 (19–83)
3-part	9 (33.3)	58.8 (24–86)
4-part	7 (25.9)	44.0 (21–76)
Head-shaft disengagement		
Residual head-shaft continuity	13 (48.1)	60.8 (19–86)
Head completely disengaged from shaft (100% translation)	14 (51.9)	47.4 (21–78)
Angulatory deformity of humeral head		
Varus displaced fracture	12 (44.4)	60.5 (24–86)
Valgus displaced fracture	5 (18.5)	43.0 (19–75)
None or fracture-dislocation	10 (37.0)	51.2 (24–78)

Mean active anterior elevation was 150°. Of all patients, 11 (40.7%) cases achieved internal rotation to T7; 9 (33.3%) cases to T12; 3 (11.1%) cases to L4; and 4 (14.8%) cases to the buttock. All the patients got fracture healing, the mean CMS and DASH scores were 73.3 (range 61–86) and 45.9 (range 27–68), respectively. More details were listed in Table 2.

**Table 2:** Comparison of functional data in different age groups

Functional parameters	Young adult, Median (range)	Adult, Median (range)	Elderly, Median (range)	P value
CMS	75 (62, 86)	65 (61, 69)	76 (61, 86)	0.306
DASH	43 (30, 68)	49.5 (37, 62)	44.5 (27, 63)	0.930

A total of four patients (14.8% of 27) experienced complications. Osteonecrosis was diagnosed in one patient with 24 months follow-up, but the patient was satisfied with the overall outcomes and without any revision surgery. Loss of reduction was observed in one patient and without any further treatment. Stiffness was observed in two patients, one of whom declined additional treatment and the other one underwent arthroscopic release for stiffness. More details were listed in Tables 3, 4.

**Table 3:** Complications

Variable	No. (%) of Patients	Treatment	No. (%) of Patients	Final outcomes	
				CMS	DASH
Loss of reduction	1 (3.7%)	No revision	1 (3.7%)	73	59
Avascular necrosis	1 (3.7%)	No revision	1 (3.7%)	82	58
Stiffness	2 (7.4%)	No revision	1 (3.7%)	61	62
		Arthroscopic release	1 (3.7%)	75	43

**Table 4:** Complications of patients in different age groups. P was obtained from Fisher’s exact test

Complications	Young adult	Adult	Elderly	P value
Yes	1^a^	1^b^	2^a^	0.355
No	10	1	12	
Total	11	2	14	

^a^Stiffness, 1 case;

^b^Stiffness, 1 case;

^c^Loss of reduction, 1 case; Avascular necrosis, 1 case

## Discussion

This is a retrospective study of peek cage and LCP used in the treatment of displaced proximal humeral fractures. Healing was achieved in all patients with a better functional outcome and a lower complication rate. Therefore, fixation augmentation using peek cage played an important role in the treatment of displaced proximal humeral fractures. In our clinical experience, there are three major effects of peek cage as follows, (1) peek cage as volumetric filling in the medial site could prevent the humeral head collapse; (2) peek cage as a wedge construct located in the space of calcar screws or medial part of the humeral head, which provides multi-dimensional stability over LCP; (3) peek cage would not disturb the blood supply of the humeral head and provide mechanical stability that allows osteogenic tissue across the fracture site and accelerate the fracture healing.

LCP is reported as a promising treatment for proximal humeral fractures. There are several advantages of the LCP system, such as the divergent angulated configuration of locking screws, anatomic design and high rotational and angular stability [12]. However, a high complication has been reported by using LCP alone [13]. A previous study showed the use of LCP in proximal humeral fractures associated with an unexpectedly high rate of complications (36%), including screw cutout (23%), varus displacement (25%), and osteonecrosis (4%) [3]. Meier et al. reported complication of protrusion in 22% (8/36) of proximal humeral fractures using angled blade-plate [14]. Fankhauser et al. reported treating 29 proximal humeral fractures with a locking proximal humeral plate [15]. They reported breakage of one plate, four episodes of redisplacement of the fracture, two cases of partial osteonecrosis, one deep infection, and two reoperations. Researchers found that lack of medial support was the main factor associated with a higher complication and a poor clinical result [6, 16, 17]. Gardner et al. [6] investigated that medial support screws played an important role in LCP fixation, but a biomechanical study showed the use of calcar screws in humeri with varus deformity without biomechanical superiority [18], and cause a high risk of humeral head necrosis [17]. Structural autograft, allograft or additional medial plate may also be used as a ‘strut’ to increase the medial stability in which the humeral head is displaced into varus, or if there is instability due to loss of the posteromedial calcar, but some drawbacks, such as donor-site morbidity, demanding technique and neurovascular injuries [19–22], were reported. Chen et al. [23] showed that proximal humeral fractures treated by LCP with fibular allograft had a better functional outcome and a lower complication rate compared to patients treated by LCP alone. The other two clinical studies also presented similar clinical results [24, 25]. In 2013, the results at minimal follow-up of a da Vinci device combined with minimal osteosynthesis or plates were reported in a series of 69 patients [26]. Then, Russo et al. [27] retrospectively compared the use of allograft or autograft and da Vinci device in proximal humeral fractures. They obtained excellent and good results in 83.2% of patients according to the Constant and DASH scores. In 2020, the invention team of the da Vinci device evaluated perioperative, early, and late complications in 142 patients [28]. The authors concluded that the intramedullary augmentation technique improves fracture treatment with significantly good anatomic reconstruction in complex and unstable fractures.

In the present study, a peek cage is inserted through the lateral fracture line into the proximal humerus with less invasive dissection. Osteonecrosis was diagnosed in 1 (3.7%) patient with a minimum of 24 months of follow-up, which is lower than the previously reported rate of 4.3% average using an external fixator [29]. We believe that the void filler can increase the mechanical stability of fractures with metaphyseal bone loss and stabilize the medial column, the head, and the tuberosity. Besides, bone graft augmentation has been considered as an effective treatment to achieve dead space-filling and a better biomechanical environment for union. Ideal bone graft substitute should be osteoinductive, osteoconductive, biocompatible, structurally similar to bone and cost-effective [30]. Autologous bone graft is considered as the gold standard grafting material and is used for most comparative analysis. But the donor site infection and donor site-related pain were reported in some related studies. Xenogenic bone has been studied as an alternative to autogenous and allogenous grafts by our previous study [31]. The main component of xenogenic bovine cancellous used in the present study was the demineralized bone matrix (DBM) with bone morphogenic protein (BMP), which showed a proper osteogenous integration of the material in animal studies [32]. DBM provides a special environment for cell migration and proliferation. The demineralization process may decrease antigenic stimulation and increase the release of BMP. BMP simulate local undifferentiated mesenchymal cells to transform into osteoblasts, and the collagenous framework of the DBM particles allows for migration of tissue into the site that may provide an additional biochemical contribution to osteogenesis.

And several technical considerations warrant emphasis. The deltopectoral approach is advised for this technique because of the direct access to the plating zone and insertion window between the fracture lines, with less invasive dissection. Close attention must be paid to the axillary nerve. Care must be taken to protect the nerve when retracting the deltoid raphe split and inserting the implants. When the peek cage is inserted, it may be not located in the proper position. With the help of fluoroscopic view, K-wire joystick or additional elevators, the peek cage can be fine-tuned. But this new technique raises several clinical considerations. Fixation augmentation by peek cage and plate may bring about excessive rigidity and stress shielding during fracture healing. And if revision surgery were required, the inner strut could be difficult to remove. Besides, the addition of peek cage for surgical treatment may be not cost-effective, which remains a major barrier to extend and promote use of it. But considering the high reoperation rates reported with current treatment, if this technique minimizes mechanical failures and revision procedures, the cost may be offset. Overall, the cage in cage technique using peek cage that locates in a cage space formed by locking screws of LCP, which forms a crucial component providing multiple-dimensional stability. Constructs by cage buttress can decrease the incidence of screw perforation, necrosis, fracture collapse, and improving shoulder function.

The main strength of this study is that all the operations were performed by the same orthopedic surgeon (WZ), which can avoid the differences caused by different surgeons’ skills. However, this study is retrospective in nature and control group is lacking. The number of patients is relatively small. And all the data of patients are collected from a single center. Therefore, more multi-center prospective randomized controlled trials are needed to verify the usefulness of this new technology (Additional file 1).

## Conclusion

In conclusion, the present results showed that fixation augmentation using peek cage and LCP is a safe and effective way to allow an anatomic reduction and stable fixation and provide a sound biomechanical environment for union and maintenance of alignment in proximal humeral fractures. Suitable void filler in the proximal humerus for reconstructing the medial column integrity attains mechanical stability in reducing the incidence of the complications.

## Supplementary Information

**Additional file 1**. The details of data in this study.

## Data Availability

All data generated or analysed during this study are included in this published article and its supplementary information files.

## Abbreviations

Peek: Polyether-ether-ketone
LCP: Locking compression plate
CMS: Constant-Murley score
DASH: Disability of the arm, shoulder and hand
CT: Computed tomography
AP: Anteroposterior
DBM: Demineralized bone matrix
BMP: Bone morphogenic protein
