# Supplementary material for: Stable osteosynthesis of cage in cage technique for surgical treatment of proximal humeral fractures
Source: BMC Surg. 2021 May 4;21:233. doi: 10.1186/s12893-021-01235-x (PMC8094560; doi:10.1186/s12893-021-01235-x)
Supplement: Supplementary file 1 — Additional file 1. The details of data in this study. [file 12893_2021_1235_MOESM1_ESM.pdf]

The details of data in this study.

No.	age	sex	injury mechanism	Occupation	Neer fracture classification	Head-shaft disengagement	Angulatory deformity of humeral head	follow-up	CMS	DASH	active anterior elevation	complication
1	73	M	Fall	Not working/retired	2	residual head-shaft continuity	varus	12	86	27	1	0
2	34	M	Vehicle accident	Manual work	3	pletely disengaged from shaft (100% translation)	none	14	62	53	3	0
3	75	F	Fall	Not working/retired	3	residual head-shaft continuity	varus	23	63	61	1	0
4	24	F	Fall	sedentary work	2	pletely disengaged from shaft (100% translation)	none	15	75	34	2	0
5	86	F	Fall	Not working/retired	3	residual head-shaft continuity	varus	24	76	38	1	0
6	24	F	Fall	Not working/retired	3	pletely disengaged from shaft (100% translation)	none	35	75	43	4	stiffness
7	35	M	Fall	Manual work	4	residual head-shaft continuity	varus	36	62	59	2	0
8	78	M	Fall	Not working/retired	3	residual head-shaft continuity	none	42	82	30	4	0
9	45	M	Fall	Not working/retired	4	pletely disengaged from shaft (100% translation)	varus	48	61	62	2	stiffness
10	21	F	Fall	Not working/retired	4	pletely disengaged from shaft (100% translation)	vagus	48	62	36	2	0
11	34	F	Fall	Manual work	4	pletely disengaged from shaft (100% translation)	none	21	68	38	2	0
12	54	M	Vehicle accident	sedentary work	4	residual head-shaft continuity	varus	15	69	37	1	0
13	76	M	Fall	Not working/retired	4	pletely disengaged from shaft (100% translation)	none	16	75	63	1	0
14	24	M	Fall	sedentary work	2	pletely disengaged from shaft (100% translation)	varus	21	76	42	2	0
15	75	F	Fall	Not working/retired	3	pletely disengaged from shaft (100% translation)	vagus	42	78	43	2	0
16	67	F	Vehicle accident	Not working/retired	3	residual head-shaft continuity	varus	35	79	48	1	0
17	75	M	Vehicle accident	Not working/retired	2	pletely disengaged from shaft (100% translation)	vagus	14	62	46	2	0
18	62	M	Vehicle accident	Not working/retired	2	residual head-shaft continuity	none	38	61	43	3	0
19	65	F	Fall	Manual work	3	residual head-shaft continuity	varus	27	76	32	3	0
20	19	M	Fall	Not working/retired	2	residual head-shaft continuity	vagus	18	82	30	1	0
21	34	F	Fall	sedentary work	2	pletely disengaged from shaft (100% translation)	none	34	83	68	2	0
22	43	M	Vehicle accident	Manual work	4	pletely disengaged from shaft (100% translation)	varus	38	84	47	1	0
23	25	F	Vehicle accident	sedentary work	3	residual head-shaft continuity	vagus	48	86	53	1	0
24	68	F	Fall	sedentary work	2	residual head-shaft continuity	none	38	82	58	1	avascular necrosis
25	76	F	Fall	Not working/retired	2	pletely disengaged from shaft (100% translation)	varus	25	80	39	1	0
26	78	M	Vehicle accident	Not working/retired	2	pletely disengaged from shaft (100% translation)	none	15	62	49	4	0
27	83	M	Vehicle accident	Not working/retired	2	residual head-shaft continuity	varus	12	73	59	4	loss of reduction
